# Respiratory Virus Infections during the COVID-19 Pandemic Revealed by Multiplex PCR Testing in Japan

**DOI:** 10.1128/spectrum.04162-22

**Published:** 2023-02-01

**Authors:** Sho Yamashita, Satoshi Ikegame, Keita Nakatomi, Yuko Sakurai, Hiroe Shuto, Noriko Sato, Yoshihiro Mizoguchi, Maki Uehara, Nobutaka Nakashima, Isamu Okamoto, Hiroshi Koto

**Affiliations:** a Kyushu Central Hospital of the Mutual Aid Association of Public School Teachers, Fukuoka, Japan; b Department of Respiratory Medicine, Graduate School of Medical Sciences, Kyushu University, Fukuoka, Japan; Quest Diagnostics Nichols Institute

**Keywords:** SARS-CoV-2, coronavirus, COVID-19, multiplex PCR, respiratory virus, FilmArray

## Abstract

Under the strict quarantine policy imposed to combat the COVID-19 (coronavirus disease 2019) pandemic in Japan, the prevalence of respiratory infections by viruses other than SARS-CoV-2 (severe acute respiratory syndrome coronavirus 2) has been largely unknown. However, such information on viral circulation is important in order to develop better management policies that are based on scientific data. Here, we retrospectively investigated respiratory virus infections in individuals who visited a community hospital with respiratory symptoms between June of 2020 and September of 2021 with the use of the BioFire FilmArray Respiratory Panel 2.1. Virus was detected in 65 out of a total of 328 subjects, with SARS-CoV-2 (67.7%), rhino/enterovirus (18.5%), and parainfluenza virus 3 (7.7%) accounting for most of the infections. No influenza virus or respiratory syncytial virus (RSV) infections were detected. The monthly cases of rhino/enterovirus infection were highest from winter to spring, with this temporal pattern differing from that of SARS-CoV-2. SARS-CoV-2 was detected more frequently (*P* < 0.001) in subjects with cough (31/104 cases, 29.8%) than in those without cough (13/224 cases, 5.8%), suggesting that cough might contribute to the prediction of COVID-19. Our findings also suggest that testing for rhino/enterovirus and parainfluenza virus 3, in addition to SARS-CoV-2, may be important for the rigorous diagnosis of respiratory virus infections.

**IMPORTANCE** Influenza virus, RSV, adenovirus, and rhino/enterovirus were the major respiratory viruses before COVID-19 pandemic. Circulating respiratory viruses may have been affected by our strong quarantine policy during the COVID-19 pandemic. We checked the circulating respiratory viruses from our outpatients by using a multiplex PCR kit that had recently been released. SARS-CoV-2 was the most frequently detected virus, and it was followed by rhino/enterovirus and parainfluenza virus 3. No influenza virus or RSV infections were detected during our study period, suggesting that influenza virus and RSV became almost extinct. COVID-19 cases frequently experienced cough, and this frequency was statistically significantly higher than that observed in the cases without SARS-CoV-2 detection. The cough can be an indicator of COVID-19.

## INTRODUCTION

Severe acute respiratory syndrome coronavirus 2 (SARS-CoV-2) emerged in China at the end of 2019 as the viral pathogen responsible for coronavirus disease 2019 (COVID-19) (1, 2). It subsequently spread worldwide with a high aerosol-mediated infectivity (3), giving rise to a global pandemic. Despite the development of efficacious vaccines that elicit the production of protective antibodies (4), SARS-CoV-2 has acquired several mutations that allow it to evade immunity that has been conferred by earlier strains (5). In particular, the omicron variant has a higher infectivity than the previous delta and alpha strains, and it manifests a distinct antigenicity that resists immunity due to prior infection with these strains (6). The omicron strain has been responsible for an unprecedented number of COVID-19 cases in Japan, and these cases have stretched the capacity of hospitals to cope with the demand for their services.

It is important to obtain insight into the local or domestic epidemiology of SARS-CoV-2 in order to prepare for future outbreaks. It is also important to characterize the domestic circulation of other respiratory pathogens in order to implement appropriate quarantine and antigen testing policies. However, the recent clinical focus has been on checking for SARS-CoV-2 infection via polymerase chain reaction (PCR) testing, with little time or effort having been devoted to checking for other viral pathogens.

A recently released multiplex PCR panel (BioFire FilmArray Respiratory Panel 2.1) (7, 8) has allowed for the simultaneous testing for SARS-CoV-2 as well as other key respiratory pathogens. We introduced this panel at Kyushu Central Hospital in June 2020 to check for respiratory viral pathogens in individuals who visited the hospital outpatient ward with respiratory symptoms. Now, we have retrospectively analyzed the test results together with the symptoms responsible for each hospital visit. We found that rhino/enterovirus accounted for almost 20% of the viral infections during the study period and that cough was a predictor of SARS-CoV-2 detection.

## RESULTS

The study included a total of 328 subjects (184 males [56.1%] and 144 females [43.9%]) who experienced respiratory symptoms and visited our hospital during the study period. The numbers of patients in the age groups of 0 to 19, 20 to 39, 40 to 59, 60 to 79, and ≥80 years of age were 10 (3.0%), 74 (22.6%), 74 (22.6%), 100 (30.5%), and 70 (21.3%), respectively. The hospital does not have a pediatrics department, explaining the relatively low proportion of subjects younger than 20 years.

**FIG 1:**
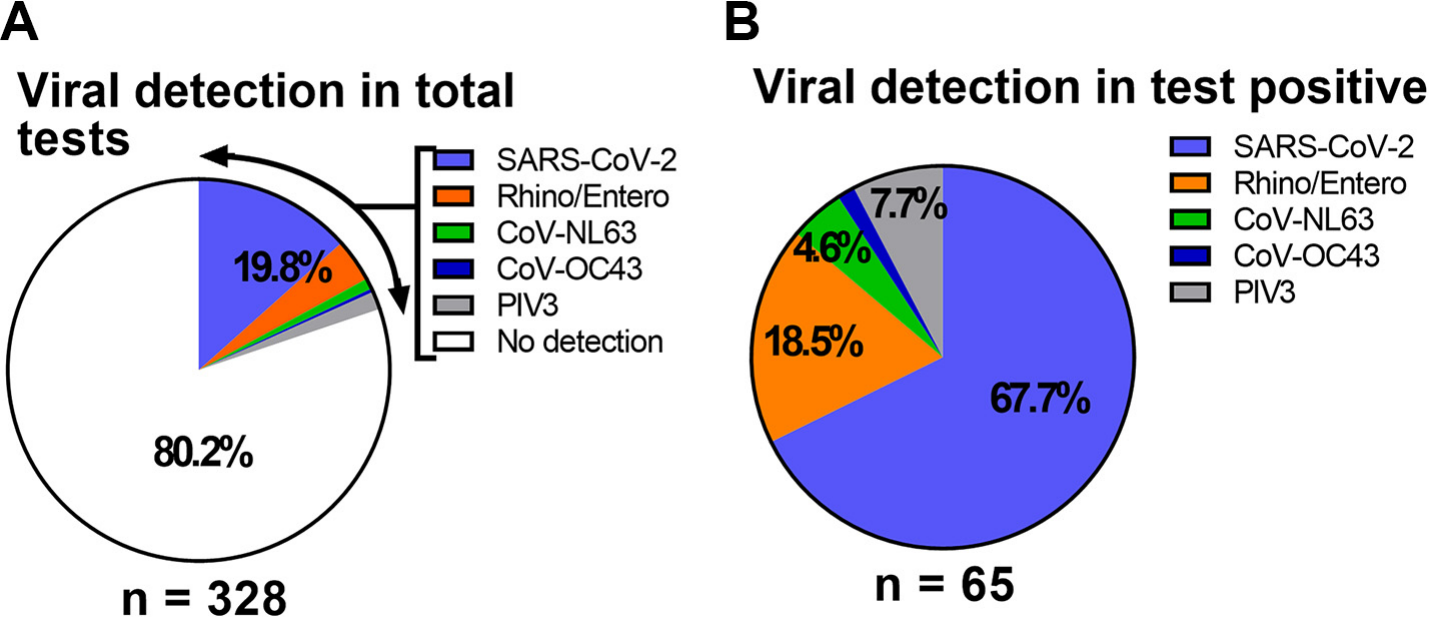
Overview of PCR test results. (A) A total of 328 subjects were tested for respiratory virus pathogens via multiplex PCR analysis, yielding 65 (19.8%) positive results. (B) Among the 65 positive cases, SARS-CoV-2 was responsible for most of the infections, followed by rhino/enterovirus, coronaviruses other than SARS-CoV-2, and parainfluenza virus 3 (PIV3).

Multiplex PCR testing revealed that 263 out of 328 subjects (80.2%) tested negative for all viral respiratory pathogens (Fig. 1A), with no subjects testing positive for bacterial pathogens. Among the 65 subjects with a positive result for viral infection, 44 (67.7%) were positive for SARS-CoV-2. We also detected rhino/enterovirus (12 subjects, 18.5%), coronavirus NL63 (3 subjects, 4.6%), coronavirus OC43 (1 subject, 1.5%), and parainfluenza virus 3 (5 subjects, 7.7%) (Fig. 1B). No influenza viruses were detected in the study subjects, suggesting that influenza did not circulate in the area served by the hospital during the COVID-19 outbreak.

**FIG 2:**
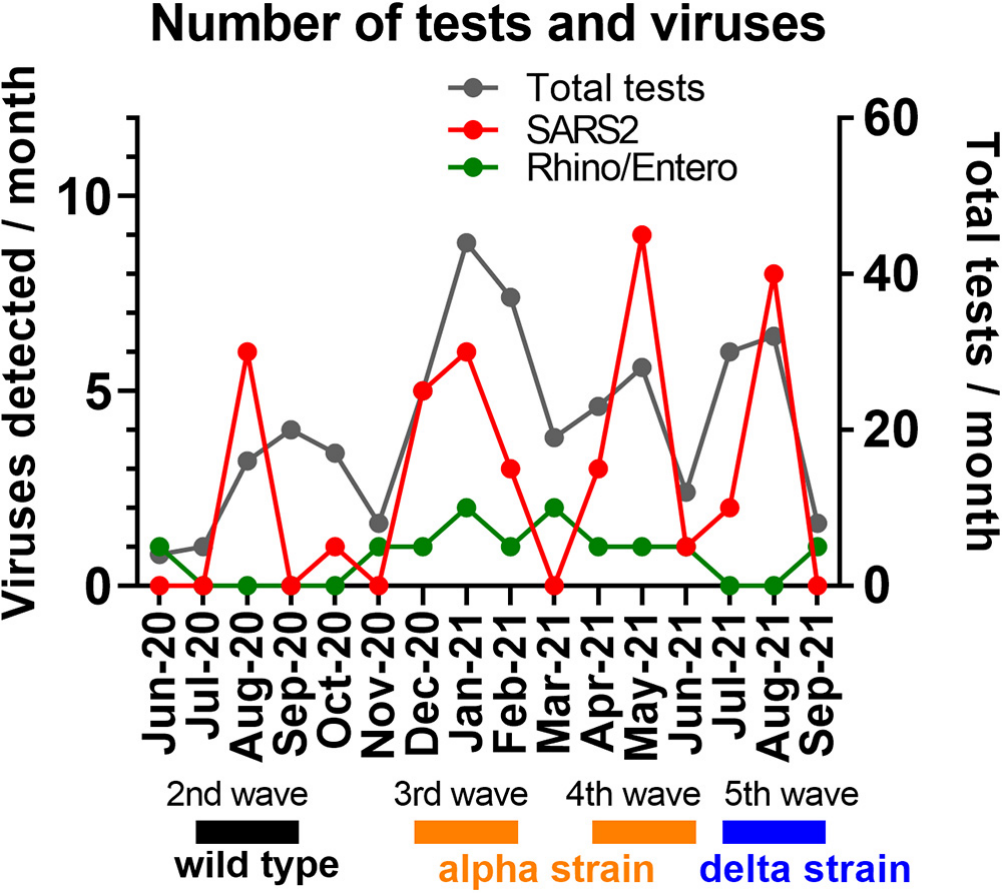
Monthly respiratory viral pathogen detection during the study period. The monthly totals of all of the PCR tests and the tests that were positive for SARS-CoV-2 or rhino/enterovirus during the study period are shown. The periods corresponding to the second through fifth national waves of SARS-CoV-2 infections that were caused by the wild-type, alpha, and delta strains are also indicated.

To examine possible seasonal trends in SARS-CoV-2 and other respiratory virus infections, we plotted the monthly viral infection cases during the study period (Fig. 2). We found four surges of SARS-CoV-2 during the study period, with the first surge corresponding to the Japanese second wave (around July of 2020). The second (December of 2020 and January of 2021) and third (around May of 2021) surges corresponded to the Japanese third and fourth waves, which were mainly caused by the B.1.1.7 strain (alpha variant) (9). The fourth surge corresponded to the Japanese fifth wave (August of 2021), which was caused by the delta variant (10). Thus, the number of SARS-CoV-2 infection cases appeared to be largely determined by the emergence and spread of new variants of concern, irrespective of the season. In contrast, rhino/enterovirus was detected throughout the winter to spring period (November to June) and thus differed in circulation pattern from SARS-CoV-2.

**FIG 3:**
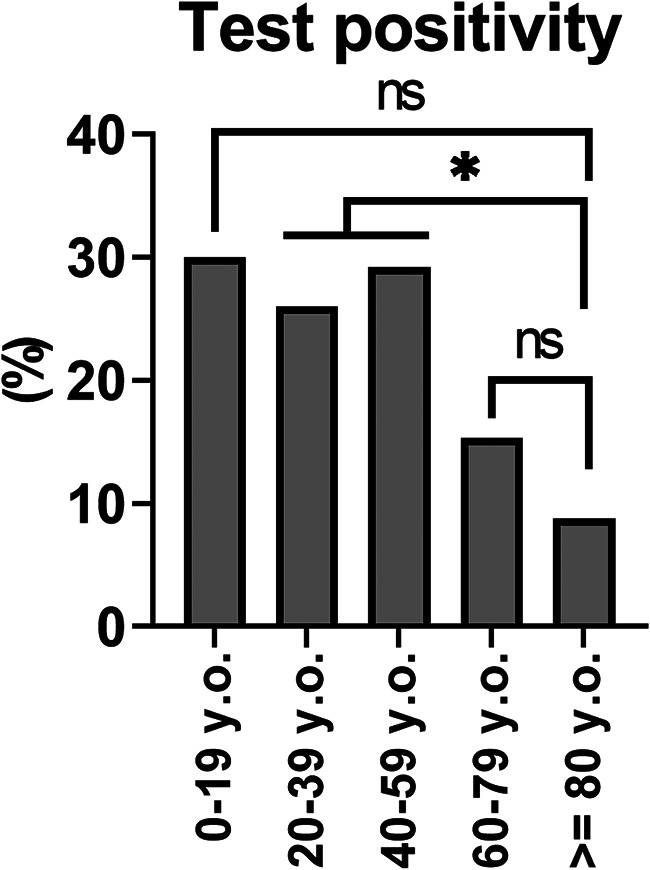
Viral test positivity, according to subject age. The overall PCR positivity rate for each age group was determined. *, *P* < 0.0125; NS, not significant (chi-square test with Bonferroni’s correction).

Next, we investigated the possible relation between age and virus isolation frequency during the SARS-CoV-2 pandemic, as was previously examined before the pandemic (11–13). The frequency of viral pathogen isolation was approximately 25% to 30% for the 0 to 19, 20 to 39, and 40 to 59 age groups, whereas it was only 15.3% and 8.8% in the 60 to 79 and ≥80 age groups, respectively (Fig. 3). The positive rate for the ≥80-year-old subjects was significantly (*P* < 0.0125) lower than those for the 20 to 39 and 40 to 59 age groups.

Finally, we examined whether certain symptoms might be associated with SARS-CoV-2 detection and might allow for the prediction of COVID-19. Table 1 shows the positivity rate for SARS-CoV-2 detection in patients with or without specific symptoms at the time of their visit to our hospital. SARS-CoV-2 positivity was higher in the presence of headache, runny nose, cough, olfactory or taste disturbance, or diarrhea (*P = *0.013, 0.025, <0.001, 0.002, and 0.007 via the chi-square test, respectively). To exclude the possible influence of confounding factors, we then performed a binomial logistic regression analysis. Cough was the only symptom found to be significantly associated with SARS-CoV-2 isolation in a manner independent of other symptoms in this analysis (odds ratio of 8.40, *P* < 0.001).

**TABLE 1:** Symptoms associated with SARS-CoV-2 detection

Symptom	Symptom present			Symptom absent				Binomial logistic regression analysis	
	SARS2 (+)	SARS2 (–)	Positive rate (%)	SARS2 (+)	SARS2 (–)	Positive rate (%)	*P* value^*a*^	Odds ratio	*P* value
Fever	40	237	14.4	4	47	7.8	0.204	1.73	0.37
Fatigue	17	79	17.7	27	205	11.6	0.142	0.86	0.71
Headache	17	61	21.8	27	223	10.8	0.013*	1.96	0.12
Runny nose	8	22	26.7	36	262	12.1	0.025*	1.35	0.57
Throat pain	12	59	16.9	32	225	12.5	0.330	0.77	0.57
Cough	31	73	29.8	13	211	5.8	<0.001***	8.40	<0.001***
Sputum	9	36	20.0	35	248	12.4	0.163	0.44	0.11
Dyspnea	8	42	16.0	36	242	12.9	0.560	0.80	0.69
Olfactory/taste disturbance	3	2	60.0	41	282	12.7	0.002**	2.51	0.39
Vomiting	1	4	20.0	43	280	13.3	0.663	2.50	0.45
Diarrhea	5	8	38.5	39	276	12.4	0.007**	4.00	0.06
Hypoxemia	5	35	12.5	39	249	13.5	0.856	1.49	0.53

a *P* values calculated by the chi-square test. *, *P* < 0.05; **, *P* < 0.01; ***, *P* < 0.001.

## DISCUSSION

Here, we evaluated the circulation of respiratory viruses, including SARS-CoV-2, with the use of the BioFire FilmArray Respiratory Panel 2.1 during the COVID-19 pandemic. This panel allows for the simultaneous detection of SARS-CoV-2, 17 other respiratory viruses, and 3 bacterial pathogens. We retrospectively analyzed outpatients presenting with respiratory symptoms, such as cough, sputum, and runny nose. Most cases of virus infection were found to be due to SARS-CoV-2, as was expected. We also detected cases of rhino/enterovirus infection, which increased in number during the winter to spring period. Thus, our findings provide important information regarding circulating viral pathogens in Japan after the implementation of a strict quarantine policy that targeted visitors from other countries (14).

We detected viral respiratory pathogens in 19.8% of individuals who visited our hospital with respiratory symptoms. A study of viral and bacterial pathogens performed with the BioFire FilmArray Respiratory Panel 2.0 in the USA in 2016 found that most viral infections were caused by human rhino/enterovirus (31.1%), RSV (12.3%), adenovirus (7.3%), and influenza virus (4.8%) and that the infection rate declined as the age of the subjects increased (11). The viral detection rate of 19.8% in our study is lower than those reported in previous studies (11, 15, 16), presumably because our study largely targeted the adult population. We also found that the virus positivity rate was significantly lower in the ≥80 age group. We speculate that the subjects in our study were highly aware that their symptoms might be due to SARS-CoV-2 and readily visited the hospital to address this concern, which might have led to a lower virus detection rate as a result of the low viral load present at symptom onset (17). It is also possible that some cases of SARS-CoV-2 infection in the community might have been diagnosed with antigen tests elsewhere, resulting in a lower test positivity rate for SARS-CoV-2 in our hospital. Even given the possibility of the underestimation of the SARS-CoV-2 infection rate, SARS-CoV-2 was still the leading cause of respiratory virus infection in our study population.

A recent study also evaluated respiratory viruses in 191 subjects with cold-like symptoms in Japan via multiplex PCR analysis and found that rhino/enterovirus (11 cases) was the most prevalent cause of infection, and they were followed by SARS-CoV-2 (8 cases), metapneumovirus (7 cases), and coronaviruses other than SARS-CoV-2 (4 cases of coronavirus 229E, 3 cases of coronavirus OC43, and 1 case of coronavirus NL63) (18). The reported virus detection rate of 20.9% (40/191 subjects) was similar to that of our study. Another study investigated viral pathogens in patients with an exacerbation of asthma or chronic obstructive pulmonary disease (COPD) between April of 2018 and March of 2020 in the Fukuoka prefecture of Japan (19), and it was thus performed in the same district as our study, just before our study period. This previous study detected human rhino/enterovirus in 32.8% and 4.5% of cases of exacerbations of asthma or COPD, respectively, as well as human metapneumovirus in 6.2% and 15.9% and parainfluenza virus in 3.1% and 9.1% of such cases, whereas it did not evaluate the involvement of SARS-CoV-2. Thus, the viral detection profiles of these two previous studies were similar to that of our study, although we did not detect human metapneumovirus. It is possible that the frequency of human metapneumovirus infection that was reported in the previous study in Fukuoka (19) reflects a specific role of this virus in the exacerbation of COPD and asthma. We did not sequence viral genomic sequences in this study, but phylogenetic/population network analyses will give us new insights about the viruses in which we are interested. Phylogenetic/population network analyses can be the next future project.

No influenza virus was detected in our study, as has recently been the case across Japan (20). It appears that domestic influenza virus essentially disappeared as a result of vaccination and the strict quarantine policy that was imposed to combat the COVID-19 pandemic. On the other hand, we did detect the circulation of rhino/enterovirus and parainfluenza virus 3, which together accounted for one-fourth of the total respiratory virus infections. The seasonal pattern of the detection of rhino/enterovirus differed from that of SARS-CoV-2. The implementation of a monitoring strategy (such as one based on the development of an antigen test) might lead to a better understanding and treatment of these viral infections.

Sneezing, sore throat, cough, and headache have been reported as common symptoms of SARS-CoV-2 infection in a human challenge trial with young subjects (21). We found that headache, runny nose, cough, olfactory or taste disturbance, and diarrhea were associated with PCR positivity for SARS-CoV-2 detection. A multivariate analysis via logistic regression found only cough to be significantly associated with SARS-CoV-2 detection, independently of other symptoms. A relation between cough and SARS-CoV-2 detection was also apparent in another recent study in Japan (22), although this previous study focused only on body temperature and cough as symptoms. Cough is a common symptom of respiratory disease and is thus not specific to SARS-CoV-2 infection. However, the presence of cough might serve as an indicator of such infection in a situation in which resources for PCR testing are lacking. A study of more than one million people in the United Kingdom found that seven symptoms (loss or change of sense of smell, loss or change of sense of taste, fever, new persistent cough, chills, appetite loss, and muscle aches) are predictive of a positive PCR result for SARS-CoV-2 (23).

The importance of our study lies in the fact that we were able to evaluate respiratory viral pathogens other than SARS-CoV-2 during the COVID-19 pandemic, which has presented different conditions for viral circulation, compared with the pre-COVID-19 era. Although our study is limited by having been performed at a single center and for only slightly longer than a year, it sheds light on the circulation of respiratory viral pathogens such as rhino/enterovirus and parainfluenza virus in addition to the marked reduction in influenza virus infection.

In conclusion, we have evaluated circulating respiratory viruses in Japan during the COVID-19 outbreak with the use of a newly released multiplex PCR kit. More than two-thirds of the detected infections were due to SARS-CoV-2, with rhino/enterovirus and parainfluenza virus 3 being the second and third most frequent viruses detected, respectively. The monthly virus isolation trend differed between SARS-CoV-2 and rhino/enterovirus, suggesting different modes of circulation in Japan. Finally, we found that cough was independently associated with SARS-CoV-2 infection.

## MATERIALS AND METHODS

We performed a retrospective analysis of patients who visited Kyushu Central Hospital between June of 2020 and September of 2021 because of symptoms and underwent a PCR test for SARS-CoV-2 and other respiratory pathogens with the BioFire FilmArray Respiratory Panel 2.1. The assay is based on nested-PCR and on the evaluation of amplified PCR products via the checking of the fluorescent intensity and the melting curve. The details of the assay are available at https://www.online-ifu.com/ITI0105. We reviewed the patients’ medical records and collected data, including their symptoms when tested, sex, age, and detected pathogens. We excluded individuals without symptoms who received a PCR test for screening purposes. The study was approved by the Institutional Review Board of Kyushu Central Hospital (protocol number rinken-306). The multiplex PCR test was performed with nasal swab specimens, according to the instructions of the manufacturer. The panel allows for the simultaneous testing for 18 viruses (adenovirus, coronavirus 229E, coronavirus HKU1, coronavirus OC43, coronavirus NL63, SARS-CoV-2, human metapneumovirus, human rhino/enterovirus, influenza A, influenza A/H1, influenza A/H1-2009, influenza A/H3, influenza B, parainfluenza virus 1, parainfluenza virus 2, parainfluenza virus 3, parainfluenza virus 4, and respiratory syncytial virus [RSV]) and for 3 bacterial pathogens (Bordetella pertussis, Chlamydophila pneumoniae, and Mycoplasma pneumoniae). The graphs were constructed with the use of GraphPad Prism 9, and the statistical analyses were performed with BellCurve for Excel, version 4.02. A *P* value of <0.05 was considered to be indicative of a statistically significant result. The Bonferroni correction (0.05 divided by the number of comparisons performed being considered to be the lower limit) was adopted when comparing multiple groups. A binomial logistic regression analysis was performed to evaluate the associations between symptoms and SARS-CoV-2 detection using BellCurve for Excel, version 4.02.

### Data availability.

All data in this study are available at https://doi.org/10.6084/m9.figshare.21256419.v2.
